# Corrigendum to “Nitric Oxide Precursors and Dimethylarginines as Risk Markers for Accelerated Measured GFR Decline in the General Population” [*Kidney International Reports* Volume 8, Issue 4, April 2023, Pages 818-826]

**DOI:** 10.1016/j.ekir.2024.05.001

**Published:** 2024-05-06

**Authors:** Nikoline B. Rinde, Inger Therese Enoksen, Toralf Melsom, Ole Martin Fuskevåg, Bjørn Odvar Eriksen, Jon Viljar Norvik

**Affiliations:** 1 Metabolic and Renal Research Group, UiT The Arctic University of Norway, Tromsø, Norway; 2 Section of Nephrology, University Hospital of North Norway, Tromsø, Norway; 3 Division of Diagnostic Services, Department of Laboratory Medicine, University Hospital of North Norway, Tromsø, Norway

The authors regret that there was a small error in the calculation of iohexol clearance (mGFR) in the third round of the Renal Iohexol Clearance Survey (RENIS-3).

The reported mean was 82.7 (15.0) mL/min/1.73 m^2^, but the revised mean (SD) should be 82.6 (14.4) mL/min/1.73 m^2^.

Corrected Tables 2 and 3 are shown below.

**Table 2:** Linear mixed model regression analyses of the associations between dimethylarginines, nitric oxide precursors and their ratios with measured glomerular filtration rate change rates (N=1407)

Independent variable	Model 1		Model 2		Model 3	
	mL/min/1.73m^2^ per year per SD∗ (95% CI)	P-value	mL/min/1.73m^2^ per year per SD∗ (95% CI)	P-value	mL/min/1.73m^2^ per year per SD∗ (95% CI)	P-value
ADMA	0.01 (-0.05 to 0.07)	0.72	0.01 (-0.05 to 0.07)	0.73	0.02 (-0.05 to 0.08)	0.59
SDMA	0.08 (0.01 to 0.14)	0.02	0.07 (0.01 to 0.14)	0.02	0.08 (0.01 to 0.14)	0.02
Arginine	-0.04 (-0.11 to 0.02)	0.17	-0.04 (-0.11 to 0.02)	0.17	-0.05 (-0.11 to 0.02)	0.16
Citrulline	-0.03 (-0.09 to 0.04)	0.42	-0.03 (-0.09 to 0.04)	0.40	-0.02 (-0.08 to 0.05)	0.59
Ornithine	-0.05 (-0.12 to 0.01)	0.10	-0.05 (-0.12 to 0.01)	0.10	-0.04 (-0.11 to 0.02)	0.17

Each row represents a separate regression model.

∗ A negative coefficient indicates a steeper decline.

Model 1: crude.

Model 2: adjusted for age, sex, and BMI.

Model 3: model 2 and adjusted for systolic blood pressure, use of angiotensin-converting enzyme inhibitors, angiotensin receptor II blockers, diuretics, calcium blockers, beta-blockers, or other antihypertensive medications (yes/no), fasting glucose, smoking status (yes/no), C-reactive protein and albumin creatinine ratio.

Abbreviations: ADMA, asymmetric dimethylarginine; Arg, arginine; OR, odds ratio; SD, standard deviation; SDMA, symmetric dimethylarginine.

**Table 3:** Associations of biomarkers with accelerated GFR decline defined as the 10% with the steepest GFR decline rate

Independent variable	Model 1		Model 2		Model 3	
	OR per SD (95% CI)	P-value	OR per SD (95% CI)	P-value	OR per SD (95% CI)	P-value
ADMA	1.10 (0.93-1.31)	0.26	1.12 (0.93-1.35)	0.23	1.10 (0.89-1.35)	0.37
SDMA	0.94 (0.79-1.13)	0.51	1.13 (0.91-1.40)	0.28	1.22 (0.97-1.55)	0.09
Arginine	1.27 (1.07-1.50)	0.007	1.17 (0.97-1.40)	0.09	1.04 (0.85-1.27)	0.72
Citrulline	1.25 (1.05-1.48)	0.010	1.44 (1.18-1.75)	<0.001	1.35 (1.09-1.68)	0.007
Ornithine	1.35 (1.15-1.58)	<0.001	1.34 (1.12-1.60)	0.001	1.21 (0.99-1.48)	0.07

Each row represents a separate regression model.

Model 1: crude.

Model 2: adjusted for age, sex, BMI, and baseline GFR.

Model 3: model 2 and adjusted for systolic blood pressure, use of angiotensin-converting enzyme inhibitors, angiotensin receptor II blockers, diuretics, calcium blockers, beta-blockers, or other antihypertensive medications (yes/no), fasting glucose, smoking status (yes/no), C-reactive protein and albumin creatinine ratio.

Abbreviations: ADMA, asymmetric dimethylarginine; Arg, arginine; GFR, glomerular filtration rate; OR, odds ratio; SD, standard deviation; SDMA, symmetric dimethylarginine.

The authors would like to apologize for any inconvenience caused.

DOI of original article: https://doi.org/10.1016/j.ekir.2023.01.015

